# Round the Hospitals

**Published:** 1918-05-25

**Authors:** 


### ROUND THE HOSPITALS.

It may be useful to point out that, as the Voting Papers sent for the election of members of the Council could not be opened except in the presence of the scrutineers after the day of election, any letters enclosed have necessarily not been answered as yet. On another page will be found a list of the papers which will be read in the course of the Conference. On the evening of June 6 Miss Gullan, sister-tutor of St. Thomas's Hospital, should have an overflowing and most intelligent audience for her address on "The Higher Education of Nurses with Relation to Applicants for Training." Miss Gullan is a university graduate, and amongst the ablest instructors of nurses the country possesses. On Friday, June 7, there will be papers in the morning on "The Advantages and Disadvantages of Preliminary Training for Intending Probationers" and "The Payment of Nurses in Training," by Miss Cummins, matron of the Royal Infirmary, Liverpool; and in the afternoon on "The Position of the Trained Nurse in Relation to the Work of Social Reconstruction." The chair on Thursday evening will be taken by Surgeon-General Sir Berkeley Moynihan, F.R.C.S., on Friday morning by Lieut.-Col. D'Arcy Power, F.R.C.S., and on Friday afternoon by Miss Haldane. All the meetings will be held at the rooms of the Medical Society of London, 11 Chandos Street, Cavendish Square, at 7.30 P.M., 11 A.M., and 3 P.M. respectively The annual meeting, for members only, at which tea will be served, takes place at the Royal Society of Medicine, 1 Wimpole Street, W., on Thursday afternoon, June 6, at 3 P.M. All meetings of the Conference are open to trained nurses, and a warm invitation is extended to everybody interested in the subjects to be discussed.

Among the institutions which have revised the scale of salaries paid to the nursing staff we are glad to record the case of the Royal Infirmary, Sheffield. Prior to the war the time-honoured theory that first-year probationers were sufficiently paid by the instruction, board, and lodging they received was in full force. From the institution point of view there is much to be said for it. The drawback is that it does not appeal to the young women of twenty-three whom the matron wants in the wards. The probationers' salaries have been raised since war began from £5, £10, £15, £25, to £10, £15, £20, £30. Sisters who formerly received £30-£35 now begin at £40 and rise by £5 a year to £50. The salaries of sisters of special departments are higher and are each considered on their merits. The additional cost to the hospital is small in comparison with the benefits secured by removing a sense of injustice from a body of women whose reputation is deservedly high in the county by reason of their high standard of training and character. Without such extra expenditure on the nursing department it is certain that the managers would have been compelled to watch the gradual deterioration of a training-school it has taken more than a generation of tireless labourers to build up.

We hope that the trend of opinion in favour of raising the salaries of nurses may not pass over the needs of the V.A.D.s in this respect. The urgency of the matter can be shown by two simple facts. The first is the difficulty experienced by many in the first instance in providing their own uniforms, seeing that an expenditure of £10 or so is a serious encroachment on future earnings. The second is the kindred difficulty in the matter of holidays. The salaries paid are so small, in fact, that these posts can hardly be accepted by persons without some private means. Salaries which are insufficient to allow something to be put aside for holidays are manifestly inadequate. Those V.A.D.s who have no friends with whom they can stay—and at present all visits are awkward because of food restrictions—are placed in a very difficult position. Many are actually unable to take the holiday which they have earned and which their work requires. This is a most unsatisfactory position of affairs, the seriousness of which is masked by the existence of those workers whose private means enable them to live upon their inadequate salaries. Every matron suffers under the staleness which afflicts her subordinates when a holiday is long overdue, and steps ought to be taken to rectify this defect in the system.

Both in this country and in America war is obliterating certain long-cherished convictions about nurses. One that has had to disappear, at any rate for a time, is the prejudice against nurses trained in small hospitals. The chiefs of Q.A.I.M.N.S.R. no longer stipulate that their nurses must produce certificates of training from hospitals with 100 beds. They are prepared to receive applications from nurses trained in hospitals with as few as forty beds. In America the Red Cross nursing authorities at first ruled all the smaller hospitals out of court and tried to limit the service to members of the State Association. This exclusiveness has had to give way to the exigencies of the war requirements, and many women on both sides of the Atlantic, whose misfortune rather than fault it has been to find themselves disqualified for patriotic work, have now come into their own. We have never yet heard it disputed that excellent nurses are turned out by the smaller training-schools, even though the opportunities afforded in these establishments may be comparatively limited.

One after another the Dominions are forming Voluntary Aid Detachments for use in the hospitals staffed by their own nurses. Australia is the latest to join in this movement, and Lady Robinson, wife of the Agent-General for Queensland, is now inviting applications from Australian women for a detachment of which she is commandant. At least 100 women over eighteen are required as wardmaids, storekeepers, pantrymaids, clerical workers, and

**ROUND THE HOSPITALS—(*continued*).**

motor drivers. Considering the very large number of Australian women in this country we cannot doubt these places will speedily be filled.

THE Red Cross rally during the past week has been undertaken in the right spirit, and ought to bring in twice as many recruits to the Service as are wanted. The clergy were asked to bring the needs of the auxiliary hospitals in respect of voluntary workers before their congregations, and the cinemas consented to arrange a pause of four minutes during which speakers addressed the audiences, and appealed to those present to offer themselves. A leaflet was also prepared for widespread distribution, giving particulars of the conditions of the Services for which candidates are needed. This is going the right way to work. London has still far too many women standing idle in the market-place for want of zeal on the part of those who hire, and many of them seem to satisfy their patriotism by nothing more strenuous than taking turns occasionally at flag-selling.

THE Devonshire Nursing Association has promulgated a new Pension Scheme with the object of enabling the district nurses to make some provision for their future. The nurses undertake to serve the Association for three years at a fixed salary in return for their training expenses, and when this time expires they are invited to join the Pension Scheme. It is proposed that each nurse shall consent to pay in 1s. a week, to be deducted from her salary, and the Association undertakes to double this contribution. The resulting benefit to the nurse, whenever she may decide to leave the Association and withdraw the amount due to her, is shown in a table from which it appears that at the end of her fifth year she would be entitled to £25, at the end of her tenth year to £75, and at the end of her twentieth year to £185 12s. 6d. It appears to be a well-thought-out and beneficial scheme, but we should have preferred a scheme affiliated with the Royal National Pension Fund for Nurses and showing the benefit in terms of an annuity as well as in terms of hard cash. The advantage of contributing automatically a small weekly sum is doubled if the nurse is drawn on to make the Fund her savings bank and increase her payments as years go on. What, after all, would a nurse be able to do with £185 after twenty years (or twenty-three years if the preliminary period be reckoned in) of district work?

A NEW departure of considerable interest has been made by the London County Council in establishing a centre for remedial exercises under the management of a local committee in Hampstead, to which selected children from schools in the district may be sent when errors in growth shall be manifested. Treatment will be given twice a week by a qualified instructress, with a view to curing or mitigating pronounced deformities which may be beyond the skill of the ordinary qualified teachers. There can be no doubt that many apparently incurable conditions will yield to remedial exercises applied while the muscles are still supple. The nurse's part in the campaign against chronic invalidism and deformity in children grows daily more full of hope and interest. But with this new current of energy in the eradication of disease comes a demand for ever-increasing efficiency on the part of nurses. It is significant to find the Hertfordshire County Health Visitor stating that while school nursing has been carried through during 1917 by sixty-six local nursing associations, the value of the work accomplished shows great variations. In certain districts visits are piled up to look well on paper, but few defects are reported, and the cases discovered at the medical inspections are not followed up. The nurse ought to observe defects and talk over with the mother any habits or modes of feeding she divines to be injurious to the child. Her training should fit her to notice defects almost by instinct and to seek at once for their cause, but for various reasons it is not always directed to this end.

CORNISH women are not coming forward in sufficient numbers to be trained for district and maternity work, and Cornish babies are dying in consequence. That is the gist of the Chairman's remarks at the annual meeting of the Cornwall Nursing Association, recently held at Truro. Many parts of the county have still no nurse. Many districts find immense difficulty in replacing their nurses when for any reason they leave. The difficulty of obtaining candidates to take a year's training is acute. And meantime Cornwall loses 500 babies a year, who might be saved if they were properly cared for. This is not to depreciate the admirable work carried out by the Cornwall Nursing Association, but it needs greater enthusiasm, more money, more volunteers. The claims of the Nursing Service are not being sufficiently advertised. Other competing organisations are allowed to secure the women who ought to be in training for nurses, and unless a more forward policy in this respect is speedily adopted the Cornish nurses will soon begin to diminish in number instead of being doubled. The committee, in our judgment, should take steps to have the claims on earnest-minded women of the District Nursing Service urged in the pulpits of churches and chapels, and brought to the attention of school-teachers throughout the Duchy. In these days it will not do to wait until nursing candidates find out the need and offer themselves. They must be sought out.

THE Women's Co-operative Guild at Hampstead has issued a well-written brochure containing considerations and suggestions as regards midwifery services which can be obtained from Miss Llewellyn Davies, 28 Church Row, Hampstead, price 3d. The Guild advocates a Public Health Service of Midwifery, paid either a yearly salary or a fee per case by the Local Public Health Authority, the Service being free to all who desire to avail themselves of it. They also advocate improved conditions of training, to include "more varied experi-

**ROUND THE HOSPITALS**—*(continued).*

ence of cases and of general nursing, and a period of practice under supervision," and they urge that inspectors and teachers of midwives should possess higher qualifications than the midwives, and that the powers of inspection should be extended. The shortage of midwives in certain districts, coupled with the steadily diminishing proportion of women who take the C.M.B. certificate with a view to practising as midwives, renders some alteration of existing conditions in the Midwifery Service imperative. But we are not yet convinced that free midwifery is desired or desirable.

Of the 500 children born every year in Shipley half die before reaching the age of one year. This holocaust of infants goes on at a curiously unequal rate in the different wards of the city, the North Ward showing an average infant mortality of 127, while in the West Ward (why does money always drift towards the west?) it is only seventy-two. The Local Government Board believes that the appointment of a whole-time health visitor would help to rescue some of these dying babies; but Shipley thinks they are not worth the money, and has rejected a proposal to appoint a visitor by eleven votes to three, under the plea that "this is not the time." The average of intelligence possessed by the councillors who voted against the proposal may be guessed by the remark of Mr. T. F. Doyle, who, in the teeth of statistics, has put on record that "he felt sure that the infantile death-rate in Shipley was a low one."

The committee of the Trowbridge Cottage Hospital, at their recent quarterly meeting, received a letter of resignation from Miss Smith, who has been matron at the hospital for four years. The clerk explained that Miss Smith was anxious to leave on May 22 with a nurse who had been her inseparable companion, and that the matron was much upset when he saw her, and evidently found her responsibilities too great. A selection from candidates had been made. The Rev. P. A. Nash suggested that an expression of appreciation should be made formally to Miss Smith at the end of the meeting, and this was accordingly done. We have often had occasion to note the exacting nature of the matron's duties in cottage hospitals, and we fear Miss Smith's health has suffered in the struggle for efficiency, rendered more difficult in both large and small establishments during the war.

We learn that Miss Macmillan, of the Borough Hospital, Birkenhead, is co-secretary with Miss Constance Worsley, who, as announced in the report of the College of Nursing published in our issue of May 11, has been appointed representative and secretary of the Liverpool Centre of the College. The dual appointment is likely to prove no sinecure, since Liverpool has many things to accomplish in the near future, and there must be an ample sphere for both secretaries. Indeed, it may be surmised that only by a division of labour could either of these busy women have accepted office.

The cause of total abstinence has many adherents among nurses who are in a position both in private and district work to watch much mental and physical deterioration through the abuse of alcohol, and frequently believe it to be their duty to abstain, as an example to others, altogether from its use. An interesting meeting of the Nurses' Total Abstinence League was held on May 11 at the London Temperance Hospital, when addresses were given by Dr. Ironside and Captain Evans. The former gave a brief account of her thirteen years' stay in Persia, where, except among the *nouveaux riches*, alcohol is a dead letter. The latter appealed to nurses, as women who were coming into their own, "to help the men to put things right." So much good has resulted during the last two years from the restrictions introduced in the sale of alcohol that it is hardly possible the old *laissez-aller* system, which lent total abstinence its strongest plea, can ever return. With England a sober nation the task of nurses, and more particularly of mental nurses, will be lightened to an extent it is hardly yet possible to realise.

The Preparatory Nursing Course advertised to take place this summer in Vassar College, in New York State, is an innovation of a remarkable character. It originated in the desire to shorten the three-year course for "graduates" to two years and three months, and thus stimulate the supply of war nurses. Although this is entirely contrary to English theories, there are features about the Preparatory Course at Vassar which are well worth studying. The idea is to provide the woman who has taken her degree with an intensive course of preparatory training, so that when she enters hospital she may be earlier of use than the pure novice. Its organisers deprecate the idea that they are arranging popular short-cut courses for amateurs. There are to be two terms of six weeks each, demanding from six to eight hours daily in class, laboratory, demonstration-room, and library. The subjects of study are to be anatomy, physiology, chemistry, bacteriology, hygiene, sanitation, nutrition and cookery, elementary materia medica, elementary nursing and hospital economy, and the historical and social aspects of nursing. Many well-known doctors, matrons, and inspectors of nurses have promised their co-operation, and the whole has been planned by the Committee on Nursing of the General Medical Committee on National Defence. When will the time come for Newnham or Girton to regard preparation for the nursing profession as worthy of their attention, and so arrange a summer course for such of their students as desire to take training as probationers?

**It is our invariable practice to pay for all items of news which we may publish. The minimum payment is 5s. Paragraphs of about twenty lines in length—that is to say, of 150 words or less—are preferred. Contributions should be addressed to The Editor, The Hospital, 28 and 29 Southampton Street, Strand, London, W.C. 2, and, to be in time for the current week's issue, should reach him by the first post on Mondays.**

**For the Economics of Nursing A Nurse's Charter of Liberty, by "Ierne," see p. 164; Nurse Training at the London Hospital, p. 170; Matrons' and Sisters' Appointments, p. 172.**

